# Predictive model of diabetes mellitus in patients with idiopathic inflammatory myopathies

**DOI:** 10.3389/fendo.2023.1118620

**Published:** 2023-04-17

**Authors:** Qiong Nie, Li Qin, Wei Yan, Qiang Luo, Tao Ying, Han Wang, Jing Wu

**Affiliations:** ^1^ Department of Geriatrics, The Affiliated Hospital of Southwest Jiaotong University, The Third People's Hospital of Chengdu, Chengdu, China; ^2^ The Center of Gastrointestinal and Minimally Invasive Surgery, Department of General Surgery, The Affiliated Hospital of Southwest Jiaotong University, The Third People's Hospital of Chengdu, Chengdu, China; ^3^ Department of Cardiology, Third Xiangya Hospital, Central South University, Changsha, China; ^4^ Department of Cardiology, The Affiliated Hospital of Southwest Jiaotong University, The Third People's Hospital of Chengdu, Chengdu, China

**Keywords:** diabetes mellitus, idiopathic inflammatory myopathies, nomogram, predictive model, cardiovascular diseases

## Abstract

**Objectives:**

Cardiovascular diseases are the common cause of death in patients with idiopathic inflammatory myopathies (IIMs). Diabetes mellitus was associated with higher cardiovascular mortality, but few studies focused on the risk of diabetes mellitus in IIMs patients. Our study is aimed at developing a predictive model of diabetes mellitus in IIMs patients.

**Methods:**

A total of 354 patients were included in this study, of whom 35 (9.9%) were diagnosed as new-onset diabetes mellitus. The predictive nomogram was drawn based on the features selected by least absolute shrinkage and selection operator (LASSO) regression, univariate logistic regression, multivariable logistic regression, and clinical relationship. The discriminative capacity of the nomogram was assessed by C-index, calibration plot, and clinical usefulness. The predictive model was verified by the bootstrapping validation.

**Results:**

The nomogram mainly included predictors such as age, gender, hypertension, uric acid, and serum creatinine. This predictive model demonstrated good discrimination and calibration in primary cohort (C-index=0.762, 95% CI: 0.677-0.847) and validation cohort (C-index=0.725). Decision curve analysis indicated that this predictive model was clinically useful.

**Conclusions:**

Clinicians can assess the risk of diabetes mellitus in IIMs patients by using this prediction model, and preventive measures should be taken early for high-risk patients, ultimately reducing the adverse cardiovascular prognosis.

## 1 Introduction

Idiopathic inflammatory myopathies (IIMs) are a group of rare autoimmune diseases characterized by chronic inflammatory infiltration of the skeletal muscle and proximal muscle weakness, which affect approximately 14.0 to 17.4 per 100,000 person-years (1). Multiple organs are involved in patients with IIMs, including skin, lungs, heart, and gastrointestinal tract, etc. Although the overall survival rate of patients with IIMs has improved, cardiac involvement remains a poor prognostic factor that as a cause of death has been reported in 10-20% of IIMs patients (2–4). Numerous studies demonstrated that the risk of cardiac involvement in patients with IIMs was higher than that in the general population, which was the same as other connective tissue diseases (CTD) (5, 6). Moreover, diabetes mellitus, a well-known traditional risk factor for cardiovascular events, was associated with higher cardiovascular mortality in patients with CTD, such as systemic lupus erythematosus (SLE) and rheumatoid arthritis (RA) (7, 8). However, the underlying mechanisms and pathogenesis of diabetes mellitus in these diseases remained unknown. Limited evidence revealed that inflammation, insulin resistance and pancreatic β cell dysfunction may play an important role in the process (8–10). More recent studies showed that diabetes mellitus in IIMs patients was not uncommon, as it occurred in about 4.2% to 29% of patients, whose prevalence was also higher than that in age- and sex-matched healthy controls (11–13). Despite the prevalence of diabetes mellitus in IIMs patients was high, it was often ignored the presence and onset of diabetes mellitus by clinicians. Of note, Yu et al. pointed out that diabetes mellitus was positively associated with mortality in patients with polymyositis and dermatomyositis (HR=2.57, 95%CI: 1.38-4.80, *P*< 0.0001) (9). In addition, patients with diabetes mellitus not only reduce the quality of life but also may lead to serious complications that increase medical expenses (14, 15). Therefore, it was crucial to predict the risk of developing diabetes mellitus in IIMs patients and then take preventive measures early for high-risk cases, and ultimately reduce the adverse cardiovascular prognosis.

To data, some models and clinical nomograms have been developed to predict the incidence of diabetes mellitus, but most of them are focused on the general population and rarely involved in subjects with autoimmune diseases. As far as we know, no study has been conducted to predict the incidence of diabetes mellitus in IIMs patients. Hence, the purpose of the current study is to establish an effective prediction model for diabetes mellitus based on demographic and clinical features of IIMs patients.

## 2 Materials and methods

### 2.1 Study population and follow-up evaluation

We conducted a retrospective cohort study including patients with IIMs who underwent a regular follow-up at the Third People’s Hospital of Chengdu between January 2010 and December 2020. The diagnosis of IIMs was determined by experienced clinicians according to the criteria of Bohan and Peter (16). All participants received periodical follow-ups and clinical examinations (at least once every three months) during the study period. The follow-up time was defined as the time from the onset of IIM to the date of diagnosis of diabetes mellitus or the last visit, whichever occurred first. Patients were not included if they had previous history of type 1 or type 2 diabetes, malignant tumor, infectious diseases, hyperthyroidism, congenital heart disease, myocardial infarction, heart failure, chronic obstructive pulmonary disease, severe hepatic and renal insufficiency, and overlap syndrome at baseline. Furthermore, subjects with irregular follow-up or incomplete data were also excluded from the study. Finally, this study included a total of 354 patients who met the above-mentioned criteria. This retrospective study was approved by the Ethic Committee of the Third People’s Hospital of Chengdu and performed in accordance with the Declaration of Helsinki (2019 S-20). All patients signed written informed consent.

Information, such as demographic characteristics, clinical manifestations, and drug administration, on each patient was collected through face-to-face interviews by trained physicians. After a 12-h fasting period, venous blood was collected in the morning from all subjects, and laboratory parameters were determined by using standard clinical laboratory techniques.

### 2.2 Diabetes mellitus assessment

During the study period, a fasting plasma glucose (FPG) ≥7.0 mmol/L, and/or self-reported diabetes can be considered as new-onset diabetes (17). Plasma glucose levels were measured on YSI glucose analyzer 2700 by the glucose oxidase method. Patients were checked at the time of diagnosis of diabetes mellitus or the last visit, whichever came first.

### 2.3 Data collection

The patients’ data were recorded in electronic medical records system during routine clinical follow-up. Demographics, clinical manifestations, laboratory parameters, and drug administration were systematically extracted from electronic medical records. Data were collected by two trained graduate students and checked by an experienced clinician.

### 2.4 Definition

In the current study, the diagnosis of overlap syndrome was based on the American College of Rheumatology (ACR) criteria for SLE (18), RA (19) and systemic sclerosis (20). Smoking was defined as having at least one cigarette per day and persisting for more than one year (21). Hypertension was defined as systolic blood pressure (SBP) ≥140mmHg and/or diastolic blood pressure (DBP) ≥90 mmHg, and/or the use of antihypertensive medication (22). The following laboratory parameters were assessed: total protein (TP, normal range: 60-83 g/L), albumin (ALB, normal range: 35-55 g/L), urea (normal range: 3.38-8.57 mmol/L), serum creatinine (Scr, normal range: 53-140 μmol/L), serum uric acid (UA, normal range: 240-490 μmol/L), triglyceride (TG, normal range: 0.29-1.83 mmol/L), total cholesterol (TC, normal range: 2.8-5.7 mmol/L), high-density lipoprotein-cholesterol (HDL-C, normal range: >0.9 mmol/L), low-density lipoprotein-cholesterol (LDL-C, normal range: <4.0 mmol/L), C-relative protein (CRP, normal range: <10 mg/L), and erythrocyte sedimentation rate (ESR, normal range: <40 mm/h).

### 2.5 Statistical analysis

All data mainly including baseline characteristics, clinical manifestations, laboratory parameters, and drug administration were expressed as count (%) or mean ± SD. All statistical analyses were conducted by using the R software (Version 4.1.0; https://www.R-project.org). A *P*-value of less than 0.1 (two-tailed) was considered statistically significant.

The least absolute shrinkage and selection operator (LASSO) regression was a punitive regression method, which estimated the regression coefficient by maximizing the logarithmic likelihood function and limited the sum of the absolute values of the regression coefficients (23). And the LASSO regression removed unnecessary covariates, which was applied to the reduction of high dimensional data. In this study, we first selected the most important variables related to diabetes mellitus in patients with IIM by using the LASSO regression model. Then, the features selected by the LASSO regression were used for univariate logistic regression and multivariable logistic regression analysis. The features were considered as odds ratio (OR) having 95% confidence interval (CI) and as *P*-value. The development of predictive model for diabetes mellitus in IIMs patients was based on the results of the LASSO regression, multivariable logistic regression, and clinical relationship.

The nomogram was utilized to show the risk prediction model of new-onset diabetes mellitus in patients with IIMs. The prediction model was validated from three aspects: discrimination ability, calibration ability, and clinical usefulness. Harrell’s C-index was used to evaluate the predictive accuracy of the nomogram (24). The C-index can range from 0.5 to 1.0. The C-index of 0.5, which represented random chance and this model had no predictive value, and the C-index of 1.0, which indicated exactly the same and this model had perfect discrimination. In general, C-index >0.7 was considered to have better discrimination ability. The goodness of fit was assessed using a calibration curve, and the area under the curve (AUC) of receiver operating characteristic (ROC) was similar to the C-index. Decision curve analysis (DCA) was also conducted to evaluate the clinical usefulness of the prediction model by quantifying the net benefits for a range of threshold probabilities in the whole cohort (25). This nomogram was further validated by bootstrapping (1000 bootstrap replicates) to calculate the relatively correctional C-index.

## 3 Results

### 3.1 Patients’ characteristics

A total of 354 IIMs patients met the inclusion criteria for this study, with the mean age of 48 years (range: 18-82 years), of whom 67.2% were female. This cohort consisted of 95 patients with polymyositis, 247 patients with dermatomyositis, and 12 patients with inclusion body myositis. The median follow-up time in the current study was 6 months (ranging from 1 to 120 months). There were 35 out of 354 patients (19 female and 16 male) who developed diabetes mellitus, with the mean age of 55 years (range: 20-76 years). The mean of baseline FPG of the included subjects was 4.72 ± 0.77 mmol/L, and there was no statistical difference in FPG between patients with or without diabetes mellitus (4.87 ± 0.73 mmol/L vs. 4.70 ± 0.78 mmol/L). The detailed characteristics of patients with IIMs were presented in **Table 1**.

**Table 1:** Clinical and demographic characteristics of IIMs patients.

Characteristics	Diabetes mellitus	Non-diabetes mellitus	Total
	(n=35)	(n=319)	(n=354)
Age (mean ± SD) (years)	55 ± 14	48 ± 13	48 ± 14
Gender (n,%)			
Female	19 (54.3)	219 (68.7)	238 (67.2)
Male	16 (45.7)	100 (31.3)	116 (32.8)
FPG (mean ± SD) (mmol/L)	4.96 ± 0.71	4.71 ± 0.78	4.73 ± 0.77
Follow-up (n,%) (months)			
<6	15 (42.9)	149 (46.7)	164 (46.3)
≥6	20 (57.1)	170 (53.3)	190 (53.7)
Smoking (n,%)	3 (8.6)	23 (7.2)	26 (7.3)
Hypertension (n,%)	10 (28.6)	40 (12.5)	50 (14.1)
Dysphagia (n,%)	10 (28.6)	78 (24.5)	88 (24.9)
Myalgia (n,%)	14 (40.0)	159 (49.8)	173 (48.9)
Arthralgia (n,%)	10 (28.6)	133 (41.7)	143 (40.4)
Rash (n,%)	25 (71.4)	216 (67.7)	241 (68.1)
Lung involvement (n,%)	13 (37.1)	114 (35.7)	127 (35.9)
Gottron’s sign (n,%)	8 (22.9)	88 (27.6)	96 (27.1)
Raynaud’s phenomenon (n,%)	0 (0.0)	33 (10.3)	33 (9.3)
ESR positive (n,%)	21 (60.0)	232 (72.7)	253 (71.5)
CRP positive (n,%)	21 (60.0)	179 (56.1)	200 (56.5)
TP (n,%)			
Below normal	11 (31.4)	94 (29.5)	105 (29.7)
Normal	24 (68.6)	222 (69.6)	246 (69.5)
Above normal	0 (0.0)	3 (0.9)	3 (0.8)
ALB (n,%)			
Below normal	18 (51.4)	151 (47.3)	169 (47.7)
Normal	17 (48.6)	168 (52.7)	185 (52.3)
Urea (n,%)			
Below normal	4 (11.4)	40 (12.5)	44 (12.4)
Normal	30 (85.7)	256 (80.3)	286 (80.8)
Above normal	1 (2.9)	23 (7.2)	24 (6.8)
Scr (n,%)			
Below normal	21 (60.0)	157 (49.2)	178 (50.3)
Normal	14 (40.0)	162 (50.8)	176 (49.7)
UA (n,%)			
Below normal	19 (54.3)	98 (30.7)	117 (33.1)
Normal	16 (45.7)	221 (69.3)	237 (66.9)
TG (n,%)			
Normal	17 (48.6)	160 (50.2)	177 (50.0)
Above normal	18 (51.4)	159 (49.8)	177 (50.0)
TC (n,%)			
Below normal	2 (5.7)	21 (6.6)	23 (6.5)
Normal	27 (77.2)	246 (77.1)	273 (77.1)
Above normal	6 (17.1)	52 (16.3)	58 (16.4)
HDL-C (n,%)			
Below normal	10 (28.6)	79 (24.8)	89 (25.1)
Normal	25 (71.4)	240 (75.2)	265 (74.9)
LDL-C (n,%)			
Normal	32 (91.4)	298 (93.4)	330 (93.2)
Above normal	3 (8.6)	21 (6.6)	24 (6.8)
Antinuclear antibody positive (n,%)	21 (60.0)	214 (67.1)	235 (66.4)
Anti SSA antibody positive (n,%)	4 (11.4)	43 (13.5)	47 (13.3)
Anti SSB antibody postive (n,%)	0 (0.0)	16 (5.0)	16 (4.5)
Anti-SCL-70 antibody postive (n,%)	0 (0.0)	5 (1.6)	5 (1.4)
Anti-Jo1 antibody postive (n,%)	1 (2.9)	23 (7.2)	24 (6.8)
Use of GC (n,%)	15 (42.9)	137 (42.9)	152 (42.9)
Use of MTX (n,%)	3 (8.6)	34 (10.7)	37 (10.5)
Use of CTX (n,%)	1 (2.9)	8 (2.5)	9 (2.5)
Use of HCQ (n,%)	1 (2.9)	23 (7.2)	24 (6.8)
Use of AZA (n,%)	1 (2.9)	6 (1.9)	7 (2.0)
Use of TII (n,%)	0 (0.0)	9 (2.8)	9 (2.5)
Use of TGP (n,%)	2 (5.7)	7 (2.2)	9 (2.5)

FPG, fasting plasma glucose; ESR, erythrocyte sedimentation rate; CRP, C-relative protein; TP, total protein; ALB, albumin; Scr, serum creatinine; UA, uric acid; TG, triglyceride; TC, total cholesterol; HDL-C, high-density lipoprotein-cholesterol; LDL-C, low-density lipoprotein-cholesterol; GC, glucocorticoid; MTX, methotrexate; CTX, cyclophosphamide; HCQ, hydroxychloroquine; AZA, azathioprine; TII, tripterygium wilfordii; TGP, total glucosides of paeony.

### 3.2 Features selection

**Figure 1:**
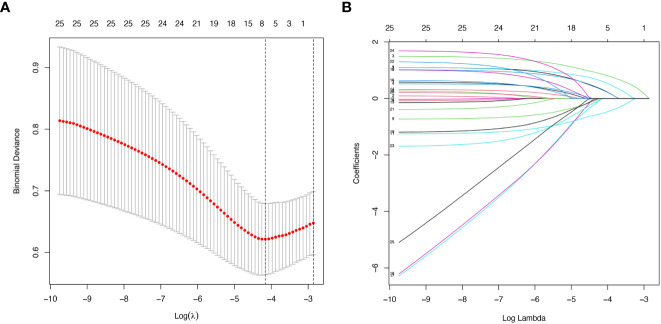
Demographic and clinical features selection using the LASSO regression model. **(A)** Optimal tuning parameter (λ) selection in the LASSO regression model used ten-fold cross-validation via minimum criteria. The binomial deviance curve was plotted versus log (λ). Dotted vertical lines were drawn at the optimal values by using the minimum criteria and 1 standard error (SE) of the minimum criteria (the 1-SE criteria). **(B)** LASSO coefficient profiles of the 35 features. A coefficient profile plot was produced against the log (λ) sequence. Vertical line was drawn at the value selected using ten-fold cross-validation, where optimal lambda resulted in 7 features with nonzero coefficients.

The LASSO regression model was applied to select the most optimal predictive features. In this study, there were 35 variables for LASSO logistic regression analysis, and 7 of them had nonzero coefficients (**Figure 1**). These 7 features included age, gender, hypertension, UA, Scr, ESR, and Raynaud’s phenomenon. Then, these 7 features were analyzed by univariate logistic regression and multivariable logistic regression, and the results pointed out that age, gender, hypertension, UA, and Scr were statistically significant between the two groups. Based on the results of LASSO regression analysis and logistic regression analysis, and clinical correlation, we selected age, gender, hypertension, UA, and Scr as predictors of diabetes mellitus in IIMs patients (**Tables 2**, **3**).

**Table 2:** Univariate logistic regression for features of diabetes mellitus.

variable	Odds ratio (95%CI)	*P*-value
Age, years		
≥60	1.5280(1.1220-2.0820)	0.0070
45-59	1.5330(0.9760-2.4080)	0.0640
18-44	1.0000(Ref.)	—
Hypertension		
Yes	3.8670(1.4640-10.2150)	0.0060
No	1.0000(Ref.)	—
Gender		
Male	2.6750(1.1890-6.0150)	0.0170
Female	1.0000(Ref.)	—
Scr		
Below normal	2.4910(1.0970-5.6530)	0.0290
Normal	1.0000(Ref.)	—
UA		
Below normal	2.7990(1.2830-6.1090)	0.0100
Normal	1.0000(Ref.)	—
Raynaud's phenomenon		
Yes	0.3320(0.0420-2.6100)	0.2950
No	1.0000(Ref.)	—
ESR		
Below normal	1.9600(0.8870-4.3350)	0.0960
Normal	1.0000(Ref.)	—

Scr, serum creatinine; UA, uric acid; ESR, erythrocyte sedimentation rate.

**Table 3:** Multivariable logistic regression model for features of diabetes mellitus.

Intercept and variable	Prediction model		
	β	Odds ratio (95%CI)	*P*-value
Intercept	-4.3077	0.0134 (0.0041-0.0386)	<0.0001
Age, years			
≥60	0.9137	2.4936(0.9027-7.1909)	0.0811
45-59	0.7651	2.1492(0.8413-5.7979)	0.1157
18-44	—	1.0000(Ref.)	—
Hypertension			
Yes	1.2247	3.4033(1.2833-8.8116)	0.0119
No	—	1.0000(Ref.)	—
Gender			
Male	1.0126	2.7529(1.2436-6.1956)	0.0129
Female	—	1.0000(Ref.)	—
Scr			
Below normal	0.8587	2.3602(1.0669-5.3914)	0.0365
Normal	—	1.0000(Ref.)	—
UA			
Below normal	0.9381	2.5550(1.1942-5.5531)	0.0160
Normal	—	1.0000(Ref.)	—

Scr, serum creatinine; UA, uric acid.

### 3.3 Development of an individualized prediction model

**Figure 2:**
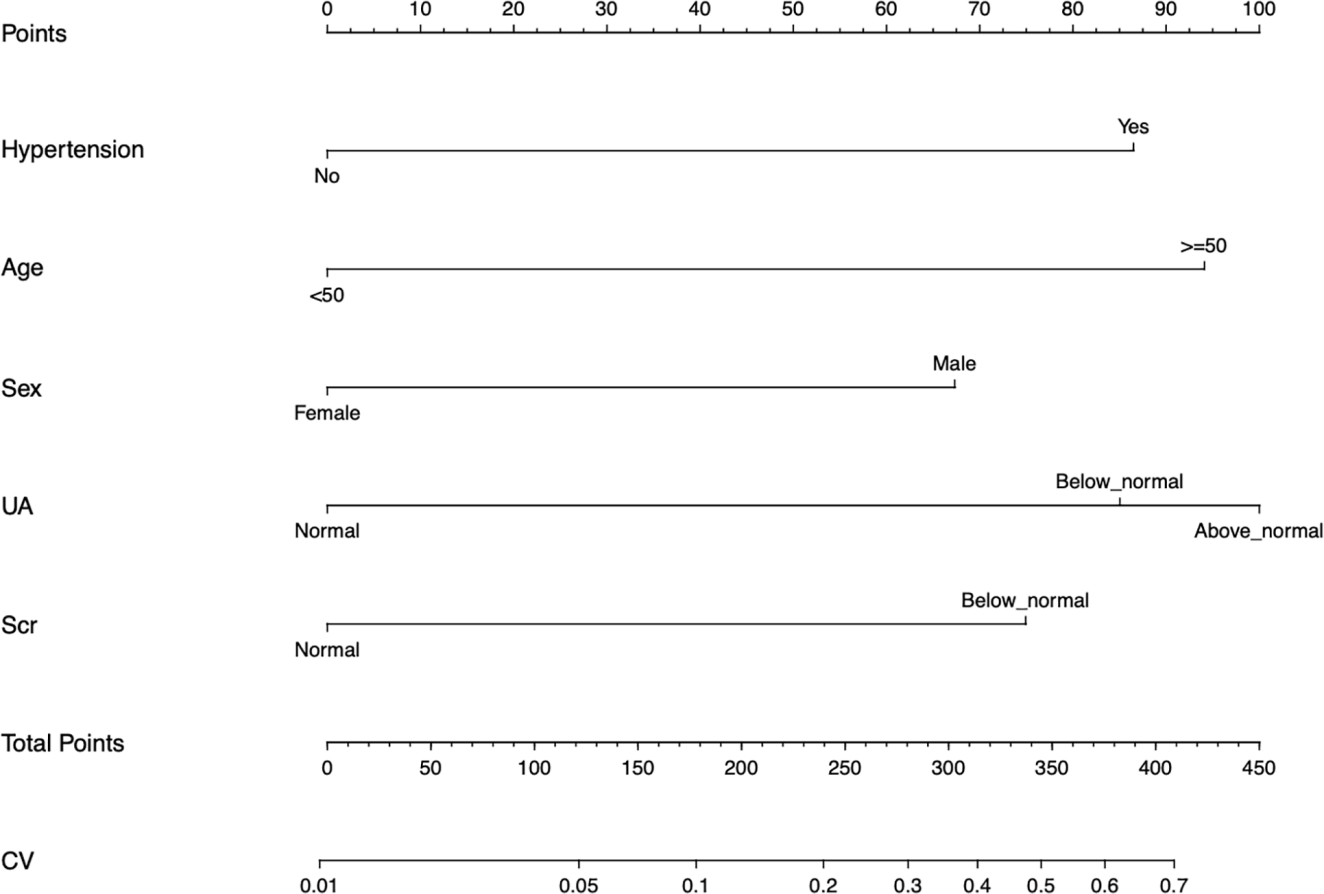
Developed the predictive nomogram of diabetes mellitus in IIMs patients. The predictive nomogram was developed in the cohort, with the age, gender, hypertension, uric acid (UA), and serum creatinine (Scr) incorporated.

As shown in **Figure 2**, the nomogram was drawn to provide a quantitative and convenient tool to predict the risk of diabetes mellitus in IIMs patients by using age, gender, hypertension, UA and Scr. The point of each predictor can be determined by drawing a vertical line to the point axis, and the total points can be calculated by summing point of each related factor in the nomogram. The higher the total points, the higher the risk of developing diabetes mellitus in patients with IIMs.

### 3.4 Validation of the nomogram of diabetes mellitus in IIMs patients

**Figure 3:**
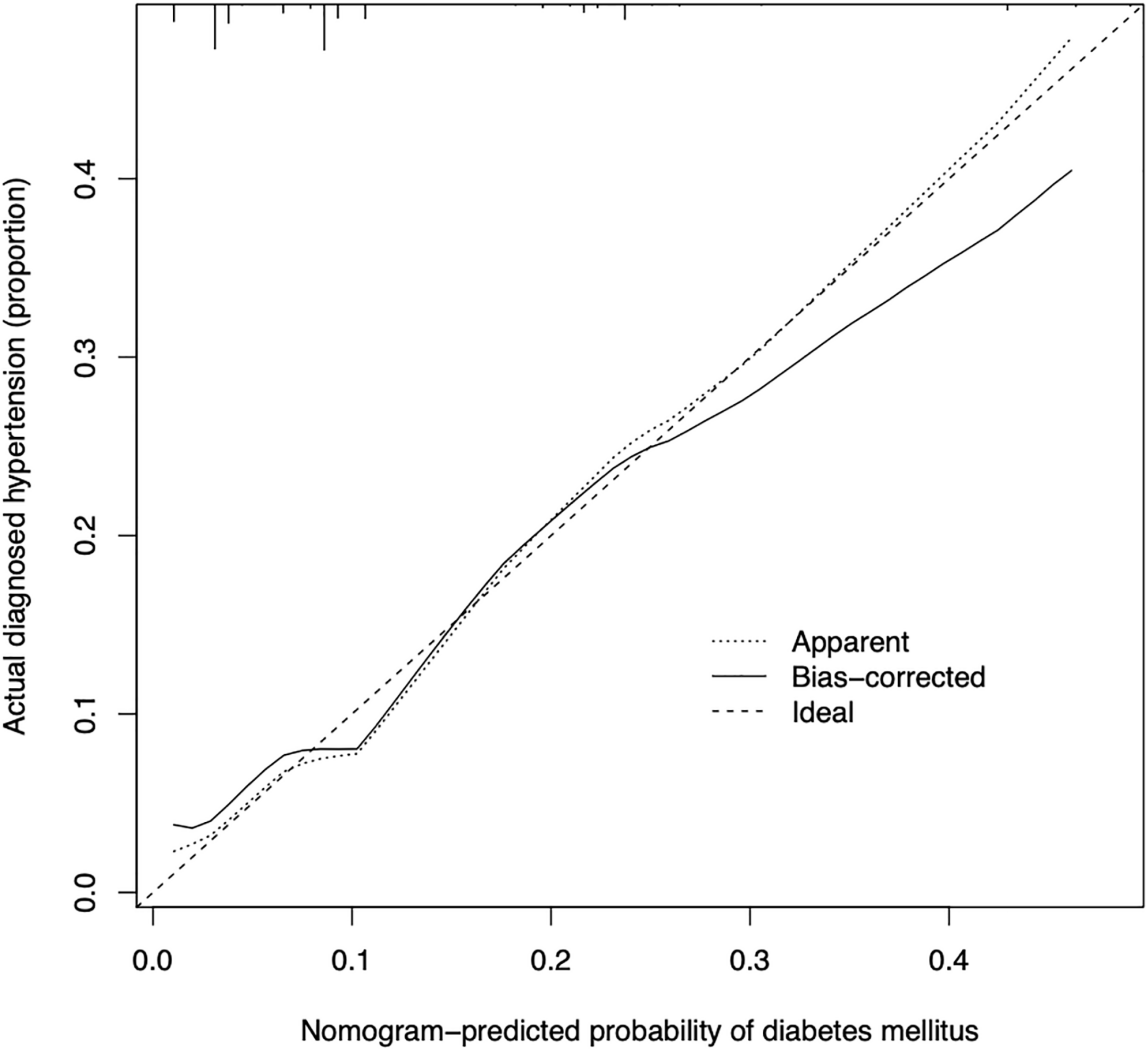
Calibration curves of the predictive nomogram of diabetes mellitus in the cohort. The x-axis represents the predicted risk of diabetes mellitus. The y-axis represents the probability of the actual diagnosed diabetes mellitus. The diagonal dotted line represents a perfect prediction by an ideal model. The solid line represents the performance of the nomogram, of which a closer fit to the diagonal dotted line represents a better prediction.

**Figure 4:**
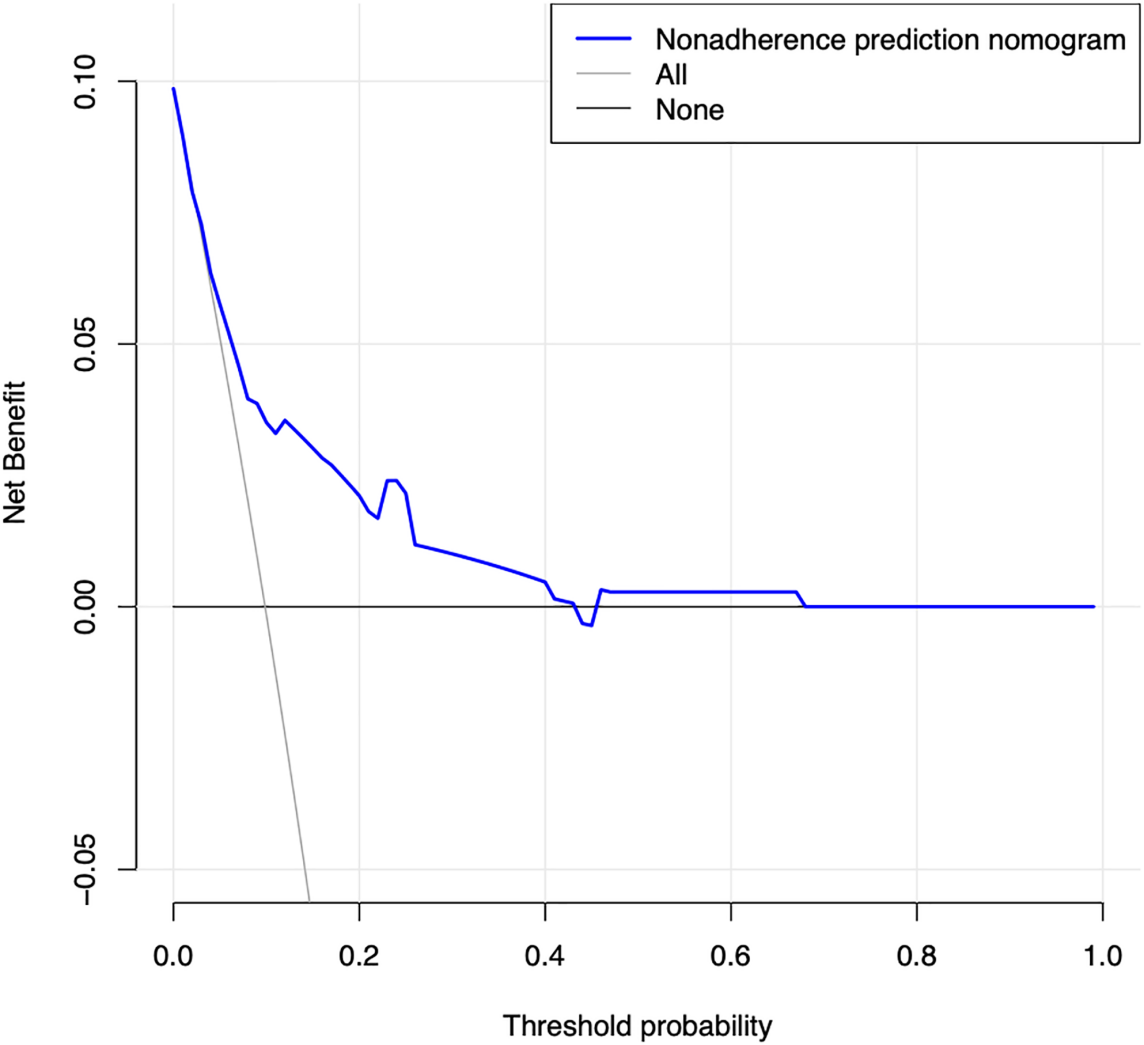
Decision curve analysis for the predictive nomogram of diabetes mellitus. The y-axis measures the net benefit. The dotted line represents the risk of predictive nomogram. The thin solid line represents the assumption that all patients have diabetes mellitus. Thin thick solid line represents the assumption that no patients have diabetes mellitus. The decision curve demonstrates that if the threshold probability of a patient and a doctor is >2% and <68%, respectively, using this nomogram to predict diabetes mellitus of IIMs adds more benefit than the scheme.

The calibration curve of the prediction diabetes mellitus nomogram in these patients presented good calibration (**Figure 3**). The C-index of this predictive nomogram was 0.762 (95% CI: 0.677-0.847) for the primary cohort, which was confirmed to be 0.725 by bootstrapping validation. The AUC value of this prediction model was 0.754 (95% CI: 0.665-0.843). DCA was used to evaluate the clinical usefulness of the predictive nomogram. From the perspective of the decision curve (**Figure 4**), if the threshold probability of a patient and a doctor was >2% and <68%, respectively, using this nomogram to predict the risk of diabetes mellitus in IIMs patients would achieve a favorable net benefit than the scheme. What’s more, the net benefit was comparable with several overlaps, on the basis of the predictive nomogram of diabetes mellitus in this range. In short, these results all suggested this model had good discrimination.

## 4 Discussion

Cardiac involvement in patients with IIMs was mostly atypical and seldom attracted the attention of clinicians. However, increasing amounts of data demonstrated that cardiovascular events were the common cause of death in IIMs. Early identification and control of cardiovascular risk factors can reduce mortality and improve the quality of life in IIMs patients. Diabetes mellitus was an indispensable risk factor for cardiovascular diseases, so it was greatly significant to identify the risk of diabetes mellitus in patients with IIMs. As a tool to evaluate the risk and prognosis of disease, nomogram has been paid more attention and applied in medical research and clinical practice. To the best of our knowledge, our study was the first to provide a relatively accurate prediction model of diabetes mellitus in patients with IIMs based on generally available clinical features. Nomogram illustrated that older age, male, hypertension, low levels of UA and Scr were more likely to develop diabetes mellitus in patients with IIMs. The C-index of the constructed nomogram and the internal validation was up to 0.762 and 0.725, respectively, which demonstrated that this predictive model had adequate discrimination and calibration. Furthermore, the decision curve analysis indicated this nomogram also had good clinical usefulness. Therefore, we think that clinicians can assess the risk of diabetes mellitus in IIMs patients by referring to this prediction model. For high-risk patients, preventive measures should be taken as soon as possible to reduce or delay the occurrence of diabetes mellitus.

The findings of our study were consistent with those of previous studies in other CTD or general population. For example, age and gender were considered to be important risk factors for diabetes mellitus, and the risk of diabetes mellitus increased with age. Similarly, a cross-sectional study demonstrated that the prevalence of diabetes mellitus increased with age, and subjects aged 60 years or older and aged 44-59 years had 2.35-fold and 2.77-fold increased risk of diabetes mellitus compared to subjects younger than 44 years, respectively (26). It may be contributed to the gradual decline of pancreatic β cell function with aging, eventually resulting in relative or absolute lack of insulin and altered glucose metabolism (27). It was worth noting that the epigenetic changes caused by aging may also affect pancreatic islets gene expression and insulin secretion. Age-related changes in pancreatic islets DNA methylation also can increase insulin resistance, impaired pancreatic β cell function, and induce diabetes mellitus (28). In addition, the prevalence of IIMs patients in women was higher than that in men, and the latter had a higher risk of developing diabetes mellitus than females that was also similar to the general population (29, 30). A study on the complications of polymyositis revealed that the prevalence of diabetes mellitus had a difference in men and women (26.1% vs 13.5%), but it was not statistically significant (P=0.201) (13). The relationship between gender and diabetes mellitus may be associated with sex steroids (31). The levels of testosterone in men decreased with the increase of age, and lower levels of serum testosterone in men were often correlated with insulin resistance, obesity and metabolic compromise (32, 33). It has been confirmed that low levels of serum testosterone in men may be applied to predict the development of type 2 diabetes (34). Additionally, there were great differences in pancreatic islets DNA methylation between genders, and extensive DNA methylation often occurred in women that can lead to increased insulin secretion, reducing the risk of diabetes mellitus (28). Hypertension was not uncommon in patients with IIMs, and the reported prevalence of hypertension in IIMs patients varied from 38.7 to 71% based on different patient selection and different definitions of disease used in the studies (12, 13). Patients with hypertension probably tended to have concomitant metabolic disorders, and researchers had identified that patients with hypertension were nearly 2.5 times more likely to develop diabetes mellitus than healthy subjects (35). In patients with hypertension, there was a 9% elevation in the risk of diabetes mellitus for each 10 mmHg increased in systolic blood pressure (36). Hypertension and diabetes mellitus often coexist and interact with each other, and their pathogenesis may be related to inflammation. Proinflammatory cytokines such as tumor-necrosis factor α (TNF-α) and interleukin-6 (IL-6) not only involved in the pathogenesis of hypertension, but also interfered with the insulin signaling pathways which were associated with insulin resistance and diabetes mellitus (37, 38). UA was mostly produced by the liver and excreted by the kidneys, and UA played a pivotal role in the antioxidant defense system in humans (39). Notably, Pitocco et al. pointed out that there was a negative correlation between UA and glycemia (r=-0.28, P=0.027), which was consistent with the result of current study (39). However, the precise mechanism of hypouricemia on the development of diabetes mellitus in IIMs patients was still poorly determined. Limited evidence demonstrated that the relationship between UA and glycemia may be contributed to the excessive production of NO caused by oxidative stress, and NO can restrict the production of UA by inhibiting the activity of endothelial xanthine oxidase activity (39, 40). Furthermore, oxidative stress often presents in the early stage of diabetes mellitus (39). To date, the study focusing on low Scr and diabetes mellitus in autoimmune diseases were rare. However, a large prospective study demonstrated that low Scr was associated with an increased risk of diabetes mellitus in the general population (41). Creatinine was a metabolite of muscle creatine and its concentration in serum was proportional to muscle mass, muscle mass was negatively related to insulin resistance and diabetes (42). Thus, it was speculated that low serum creatinine may be a predictor of diabetes mellitus (43). It was worth mentioning that whether the clinical manifestations and the treatment of IIMs were associated with the development of diabetes mellitus was still unknown. However, a study presented that hydroxychloroquine (HCQ), methotrexate (MTX) and tumor-necrosis factor inhibitors (TNFi) were related to decreased risk of incident diabetes mellitus in RA patients, and glucocorticoid (GC) was associated with increased risk of diabetes mellitus in a dose-dependent manner (44). The mechanism underlying the diabetes risk reduction with HCQ, MTX and TNFi may be attributed to the reduction of inflammatory, the improvement of glucose metabolism and pancreatic β cell function (44). Glucocorticoid increased glucose by augmenting hepatic gluconeogenesis, inhibiting glucose uptake in adipose tissue, and antagonizing insulin-mediated glucose disposal in a dose-dependent manner (45, 46). Therefore, drugs should be reasonably selected to reduce the risk of diabetes in the treatment of autoimmune diseases.

There are currently no studies on diabetes risk prediction models in connective tissue diseases; But in recent years, multivariate risk scores have been developed to predict the risk of diabetes in healthy individuals and most risk scores contain typical diabetes risk factors such as age, gender, degree of obesity, family history of diabetes and blood pressure status (47–50). In this study, a prediction model of diabetes prevalence in IIMs was developed, and the important characteristics derived included age, gender, hypertension, UA, and Scr, which were somewhat coincident and similar to the results of previous studies (48). Due to different research factors and statistical methods, different prediction models may also get different prediction findings. Previous studies mostly used univariate or multivariate analysis to obtain the results, which may be prone to multicollinearity problems. LASSO regression analysis can better resolves confound from multicollinearity (51). It builds a model with better accuracy and stability by reducing the complexity of the model. After filtering out meaningful variables, univariate and multivariate logistic regression analyses were used again to make the results more reliable and stable. The constructed model was also verified for its discrimination, calibration and clinical applicability, thus we can establish a model with better validity and applicability.

There are some limitations to this study that are worth mentioning. First, our study is a retrospective study in which individuals with incomplete data are exclude, which may lead to selection bias. Second, although this study includes a wide range of potential predictors, there are other factors that cannot be acquired, such as drinking status, family history of diabetes, and the dosage of drugs, etc, which may lead to the limited prediction power of the model. Third, the sample size of this cohort is relatively small and it is not representative of all Chinese patients with IIMs. Nonetheless, as far as we know, this is the largest prediction model focused on diabetes mellitus in autoimmune diseases. Fourth, the prediction model is not validated externally. Therefore, more prospective studies are needed to further confirm the current results and make it universally applicable.

In conclusion, we have established a relatively accurate and simple nomogram based on five risk factors, namely age, male, hypertension, hypouricemia, and low serum creatinine, to predict the risk of developing diabetes mellitus in IIMs patients. Clinicians and patients can take more necessary measures early to reduce the incidence of diabetes mellitus for high-risk patients.

## Data availability statement

The original contributions presented in the study are included in the article/supplementary material. Further inquiries can be directed to the corresponding authors.

## Ethics statement

The study was conducted according to the guidelines of the Declaration of Helsinki, and approved by the Ethic Committee of the Third People’s Hospital of Chengdu (2019 S-20) on 20 March 2021. The patients/participants provided their written informed consent to participate in this study.

## Author contributions

QN and LQ performed the study design and wrote the manuscript. QN, WY and QL performed data collection and analysis. LQ and TY conducted validation and formal analysis. HW and JW substantially revised, commented on and approved the final manuscript. All authors contributed to the article and approved the submitted version.

## References

[1] Furst DE Amato AA Iorga SR Gajria K Fernandes AW. Epidemiology of adult idiopathic inflammatory myopathies in a U.S. managed care plan. Muscle Nerve (2012) 45:676–83. doi: 10.1002/mus.23302 22499094

[2] Sultan SM Ioannou Y Moss K Isenberg DA. Outcome in patients with idiopathic inflammatory myositis: morbidity and mortality. Rheumatology (2002) 41:22–6. doi: 10.1093/rheumatology/41.1.22 11792875

[3] Schiopu E Phillips K MacDonald PM Crofford LJ Somers EC. Predictors of survival in a cohort of patients with polymyositis and dermatomyositis: effect of corticosteroids, methotrexate and azathioprine. Arthritis Res Ther (2012) 14:R22. doi: 10.1186/ar3704 22284862 PMC3392815

[4] Liu XH Feng XJ Shi JY Jia FW Liu YX Zhu YL. The quest for diagnostic approaches of cardiac involvement in polymyositis and dermatomyositis. Ann Palliat Med (2020) 9:2256–70. doi: 10.21037/apm-19-650 32648461

[5] Gupta R Wayangankar SA Targoff IN Hennebry TA. Clinical cardiac involvement in idiopathic inflammatory myopathies: a systematic review. Int J Cardiol (2011) 148:261–70. doi: 10.1016/j.ijcard.2010.08.013 20826015

[6] Opinc AH Makowski MA Łukasik ZM Makowska JS. Cardiovascular complications in patients with idiopathic inflammatory myopathies: does heart matter in idiopathic inflammatory myopathies? Heart Fail Rev (2021) 26:111–25. doi: 10.1007/s10741-019-09909-8 31867681

[7] Skielta M Söderström L Rantapää-Dahlqvist S Jonsson SW Mooe T. Trends in mortality, co-morbidity and treatment after acute myocardial infarction in patients with rheumatoid arthritis 1998-2013. Eur Heart J Acute Cardiovasc Care (2020) 9:931–8. doi: 10.1177/2048872619896069 31990203

[8] Zeng YJ Zeng FQ Dai L Yang C Lin BZ Zheng DH. Characteristics and risk factors for hyperglycemia in Chinese female patients with systemic lupus erythematosus. Lupus (2010) 19:1344–50. doi: 10.1177/0961203310375439 20693192

[9] Yu KH Wu YJ Kuo CF See LC Shen YM Chang HC. Survival analysis of patients with dermatomyositis and polymyositis: analysis of 192 Chinese cases. Clin Rheumatol (2011) 30:1595–601. doi: 10.1007/s10067-011-1840-0 21915609

[10] Danve AS Kulkarni S. Do tumor necrosis factor (TNF) inhibitors improve the glycemic control in patients with rheumatoid arthritis and concomitant diabetes mellitus? Am J Ther (2017) 24:e347–50. doi: 10.1097/mjt.0000000000000297 26103543

[11] Pi H Zhou H Jin H Ning Y Wang Y. Abnormal glucose metabolism in rheumatoid arthritis. BioMed Res Int (2017) 2017:9670434. doi: 10.1155/2017/9670434 28529957 PMC5424188

[12] Narayanaswamy AS Akhtar M Kumar N Lazar AI. Polymyositis–a review and follow up study of 24 cases. J Assoc Physicians India (1993) 41:354–6. 8005972

[13] Diederichsen LP Diederichsen AC Simonsen JA Junker P Søndergaard K Lundberg IE. Traditional cardiovascular risk factors and coronary artery calcification in adults with polymyositis and dermatomyositis: a Danish multicenter study. Arthritis Care Res (Hoboken) (2015) 67:848–54. doi: 10.1002/acr.22520 25418360

[14] Souza FH Levy-Neto M Shinjo SK. Prevalence of clinical and laboratory manifestations and comorbidities in polymyositis according to gender. Rev Bras Reumatol (2011) 51:428–83. 21952995

[15] Papatheodorou K Papanas N Banach M Papazoglou D Edmonds M. Complications of diabetes 2016. J Diabetes Res (2016) 2016:6989453. doi: 10.1155/2016 27822482 PMC5086373

[16] Bohan A Peter JB. Polymyositis and dermatomyositis (first of two parts). N Engl J Med (1975) 292:344–7. doi: 10.1056/nejm197502132920706 1090839

[17] Inzucchi SE. Clinical practice. diagnosis of diabetes. N Engl J Med (2012) 367:542–50. doi: 10.1056/NEJMcp1103643 22873534

[18] Hochberg MC. Updating the American college of rheumatology revised criteria for the classification of systemic lupus erythematosus. Arthritis Rheum (1997) 40:1725. doi: 10.1002/art.1780400928 9324032

[19] Aletaha D Neogi T Silman AJ Funovits J Felson DT Bingham CO 3rd. 2010 Rheumatoid arthritis classification criteria: an American college of Rheumatology/European league against rheumatism collaborative initiative. Ann Rheum Dis (2010) 69:1580–8. doi: 10.1136/ard.2010.138461 20699241

[20] van den Hoogen F Khanna D Fransen J Johnson SR Baron M Tyndall A. 2013 Classification criteria for systemic sclerosis: an American college of rheumatology/European league against rheumatism collaborative initiative. Ann Rheum Dis (2013) 72:1747–55. doi: 10.1136/annrheumdis-2013-204424 24092682

[21] Wang Y Wu H Liu Q Wang C Fu L Wang H. Association of CHRNA5-A3-B4 variation with esophageal squamous cell carcinoma risk and smoking behaviors in a Chinese population. PloS One (2013) 8:e67664. doi: 10.1371/journal.pone.0067664 23844051 PMC3699625

[22] Unger T Borghi C Charchar F Khan NA Poulter NR Prabhakaran D. 2020 International society of hypertension global hypertension practice guidelines. Hypertension (2020) 75:1334–57. doi: 10.1097/hjh.0000000000002453 32370572

[23] Sauerbrei W Royston P Binder H. Selection of important variables and determination of functional form for continuous predictors in multivariable model building. Stat Med (2007) 26:5512–28. doi: 10.1002/sim.3148 18058845

[24] Harrell FE Jr. Califf RM Pryor DB Lee KL Rosati RA. Evaluating the yield of medical tests. JAMA (1982) 247:2543–6. 7069920

[25] Vickers AJ Cronin AM Elkin EB Gonen M. Extensions to decision curve analysis, a novel method for evaluating diagnostic tests, prediction models and molecular markers. BMC Med Inform Decis Mak (2008) 8:53. doi: 10.1186/1472-6947-8-53 19036144 PMC2611975

[26] Wang R Zhang P Li Z Lv X Cai H Gao C. The prevalence of pre-diabetes and diabetes and their associated factors in northeast China: a cross-sectional study. Sci Rep (2019) 9:2513. doi: 10.1038/s41598-019-39221-2 30792436 PMC6385189

[27] Basu R Breda E Oberg AL Powell CC Dalla Man C Basu A. Mechanisms of the age-associated deterioration in glucose tolerance: contribution of alterations in insulin secretion, action, and clearance. Diabetes (2003) 52:1738–48. doi: 10.2337/diabetes.52.7.1738 12829641

[28] Davegårdh C García-Calzón S Bacos K Ling C. DNA Methylation in the pathogenesis of type 2 diabetes in humans. Mol Metab (2018) 14:12–25. doi: 10.1016/j.molmet.2018.01.022 29496428 PMC6034041

[29] Limaye VS Blumbergs P Roberts-Thomson PJ. Idiopathic inflammatory myopathies. Intern Med J (2009) 39:179–90. doi: 10.1159/000212374 19006486

[30] Yang W Lu J Weng J Jia W Ji L Xiao J. Prevalence of diabetes among men and women in China. N Engl J Med (2010) 362:1090–101. doi: 10.1056/nejmoa0908292 20335585

[31] Allan CA. Sex steroids and glucose metabolism. Asian J Androl (2014) 16:232–8. doi: 10.4103/1008-682x.122589 PMC3955332 24457840

[32] Jones TH. Effects of testosterone on type 2 diabetes and components of the metabolic syndrome. J Diabetes (2010) 2:146–56. doi: 10.1111/j.1753-0407.2010.00085.x 20923480

[33] Zeng QS Xu CL Liu ZY Wang HQ Yang B Xu WD. Relationship between serum sex hormones levels and degree of benign prostate hyperplasia in Chinese aging men. Asian J Androl (2012) 14:773–7. doi: 10.1038/aja.2012.32 PMC3734997 22751417

[34] Oh JY Barrett-Connor E Wedick NM Wingard DL Rancho Bernardo S. Endogenous sex hormones and the development of type 2 diabetes in older men and women: the rancho Bernardo study. Diabetes Care (2002) 25:55–60. doi: 10.2337/diacare.25.1.55 11772901

[35] Climie RE van Sloten TT Bruno RM Taddei S Empana JP Stehouwer CDA. Macrovasculature and microvasculature at the crossroads between type 2 diabetes mellitus and hypertension. Hypertension (2019) 73:1138–49. doi: 10.1161/hypertensionaha.118.11769 31067192

[36] Li X Wang J Shen X An Y Gong Q Li H. Higher blood pressure predicts diabetes and enhances long-term risk of cardiovascular disease events in individuals with impaired glucose tolerance: Twenty-three-year follow-up of the daqing diabetes prevention study. J Diabetes (2019) 11:593–8. doi: 10.1111/1753-0407.12887 PMC6618010 30556339

[37] Bautista LE Vera LM Arenas IA Gamarra G. Independent association between inflammatory markers (C-reactive protein, interleukin-6, and TNF-alpha) and essential hypertension. J Hum Hypertens (2005) 19:149–54. doi: 10.1038/sj.jhh.1001785 15361891

[38] Bastard JP Maachi M Lagathu C Kim MJ Caron M Vidal H. Recent advances in the relationship between obesity, inflammation, and insulin resistance. Eur Cytokine Netw (2006) 17:4–12. 16613757

[39] Pitocco D Di Stasio E Romitelli F Zaccardi F Tavazzi B Manto A. Hypouricemia linked to an overproduction of nitric oxide is an early marker of oxidative stress in female subjects with type 1 diabetes. Diabetes Metab Res Rev (2008) 24:318–23. doi: 10.1002/dmrr.814 18254136

[40] Cote CG Yu FS Zulueta JJ Vosatka RJ Hassoun PM. Regulation of intracellular xanthine oxidase by endothelial-derived nitric oxide. Am J Physiol (1996) 271:L869. doi: 10.1152/ajplung.1996.271.5.l869 8944732

[41] Bao X Gu Y Zhang Q Liu L Meng G Wu H. Low serum creatinine predicts risk for type 2 diabetes. Diabetes Metab Res Rev (2018) 34:e3011. doi: 10.1002/dmrr.3011 29633473

[42] Park J Mehrotra R Rhee CM Molnar MZ Lukowsky LR Patel SS. Serum creatinine level, a surrogate of muscle mass, predicts mortality in peritoneal dialysis patients. Nephrol Dial Transplant (2013) 28:2146–55. doi: 10.1093/ndt/gft213 PMC3765023 23743018

[43] Hjelmesaeth J Røislien J Nordstrand N Hofsø D Hager H Hartmann A. Low serum creatinine is associated with type 2 diabetes in morbidly obese women and men: a cross-sectional study. BMC Endocr Disord (2010) 10:6. doi: 10.1186/1472-6823-10-6 20398422 PMC2861032

[44] Xie W Yang X Ji L Zhang Z. Incident diabetes associated with hydroxychloroquine, methotrexate, biologics and glucocorticoids in rheumatoid arthritis: A systematic review and meta-analysis. Semin Arthritis Rheum (2020) 50:598–607. doi: 10.1016/j.semarthrit.2020.04.005 32480098

[45] Movahedi M Beauchamp ME Abrahamowicz M Ray DW Michaud K Pedro S. Risk of incident diabetes mellitus associated with the dosage and duration of oral glucocorticoid therapy in patients with rheumatoid arthritis. Arthritis Rheumatol (2016) 68:1089–98. doi: 10.1002/art.39537 PMC4982029 26663814

[46] Do TTH Marie G Héloïse D Guillaume D Marthe M Bruno F. Glucocorticoid-induced insulin resistance is related to macrophage visceral adipose tissue infiltration. J Steroid Biochem Mol Biol (2019) 185:150–62. doi: 10.1016/j.jsbmb.2018.08.010 30145227

[47] Allaoui G Rylander C Averina M Wilsgaard T Fuskevåg OM Berg V. Longitudinal changes in blood biomarkers and their ability to predict type 2 diabetes mellitus-the tromsø study. Endocrinol Diabetes Metab (2022) 5:e00325. doi: 10.1002/edm2.325 35147293 PMC8917864

[48] Arellano-Campos O Gómez-Velasco DV Bello-Chavolla OY Cruz-Bautista I Melgarejo-Hernandez MA Muñoz-Hernandez L. Development and validation of a predictive model for incident type 2 diabetes in middle-aged Mexican adults: the metabolic syndrome cohort. BMC Endocr Disord (2019) 19:41. doi: 10.1186/s12902-019-0361-8 31030672 PMC6486953

[49] Schmid R Vollenweider P Bastardot F Vaucher J Waeber G Marques-Vidal P. Current genetic data do not improve the prediction of type 2 diabetes mellitus: the CoLaus study. J Clin Endocrinol Metab (2012) 97:E1338–41. doi: 10.1210/jc.2011-3412 22535968

[50] Raynor LA Pankow JS Duncan BB Schmidt MI Hoogeveen RC Pereira MA. Novel risk factors and the prediction of type 2 diabetes in the atherosclerosis risk in communities (ARIC) study. Diabetes Care (2013) 36:70–6. doi: 10.2337/dc12-0609 PMC3526210 22933437

[51] Tibshirani R. Regression shrinkage and selection via the lasso. J R Stat Soc Ser B Methodol (1996) 58:267–88. doi: 10.1111/j.2517-6161.1996.tb02080.x

